# Perturbations in fatty acid metabolism and collagen production infer pathogenicity of a novel *MBTPS2* variant in Osteogenesis imperfecta

**DOI:** 10.3389/fendo.2023.1195704

**Published:** 2023-05-25

**Authors:** Pei Jin Lim, Giulio Marcionelli, Pakeerathan Srikanthan, Timothée Ndarugendamwo, Jason Pinner, Marianne Rohrbach, Cecilia Giunta

**Affiliations:** ^1^ Connective Tissue Unit, Division of Metabolism and Children’s Research Center, University Children’s Hospital Zurich and University of Zurich, Zurich, Switzerland; ^2^ Department of Clinical Chemistry and Biochemistry, University Children’s Hospital Zurich, University of Zurich, Zurich, Switzerland; ^3^ Centre for Clinical Genetics, Sydney Children’s Hospital, Sydney, Australia; ^4^ UNSW Medicine and Health, University of New South Wales, Sydney, Australia

**Keywords:** osteogenesis imperfecta (OI), extracellular matrix (ECM), fatty acid metabolism, X-linked, site-2 protease, MBTPS2, X-linked osteogenesis imperfecta

## Abstract

Osteogenesis imperfecta (OI) is a heritable and chronically debilitating skeletal dysplasia. Patients with OI typically present with reduced bone mass, tendency for recurrent fractures, short stature and bowing deformities of the long bones. Mutations causative of OI have been identified in over 20 genes involved in collagen folding, posttranslational modification and processing, and in bone mineralization and osteoblast development. In 2016, we described the first X-linked recessive form of OI caused by *MBTPS2* missense variants in patients with moderate to severe phenotypes. *MBTPS2* encodes site-2 protease, a Golgi transmembrane protein that activates membrane-tethered transcription factors. These transcription factors regulate genes involved in lipid metabolism, bone and cartilage development, and ER stress response. The interpretation of genetic variants in *MBTPS2* is complicated by the gene’s pleiotropic properties; *MBTPS2* variants can also cause the dermatological conditions Ichthyosis Follicularis, Atrichia and Photophobia (IFAP), Keratosis Follicularis Spinulosa Decalvans (KFSD) and Olmsted syndrome (OS) without skeletal abnormalities typical of OI. Using control and patient-derived fibroblasts, we previously identified gene expression signatures that distinguish *MBTPS2*-OI from *MBTPS2*-IFAP/KFSD and observed stronger suppression of genes involved in fatty acid metabolism in *MBTPS2*-OI than in *MBTPS2*-IFAP/KFSD; this was coupled with alterations in the relative abundance of fatty acids in *MBTPS2*-OI. Furthermore, we observed a reduction in collagen deposition in the extracellular matrix by *MBTPS2*-OI fibroblasts. Here, we extrapolate our observations in the molecular signature unique to *MBTPS2*-OI to infer the pathogenicity of a novel *MBTPS2* c.516A>C (p.Glu172Asp) variant of unknown significance in a male proband. The pregnancy was terminated at gestational week 21 after ultrasound scans showed bowing of femurs and tibiae and shortening of long bones particularly of the lower extremity; these were further confirmed by autopsy. By performing transcriptional analyses, gas chromatography-tandem mass spectrometry-based quantification of fatty acids and immunocytochemistry on fibroblasts derived from the umbilical cord of the proband, we observed perturbations in fatty acid metabolism and collagen production similar to what we previously described in *MBTPS2*-OI. These findings support pathogenicity of the *MBTPS2* variant p.Glu172Asp as OI-causative and highlights the value of extrapolating molecular signatures identified in multiomics studies to characterize novel genetic variants.

## 1 Introduction

The membrane-bound transcription factor peptidase site 2 (*MBTPS2*, ENSG00000012174) encodes site-2 protease (S2P), a zinc metalloprotease that plays a central role in regulated intramembrane proteolysis (RIP) (1). This process takes place on the Golgi membrane and is responsible for the activation of membrane-tethered transcription factors, including the activating transcription factor 6 (ATF6), the box B-binding factor 2 human homolog on chromosome 7 (BBF2H7), the old astrocyte specifically induced substance (OASIS) and the sterol regulatory element binding protein (SREBP). Once cleaved, the cytoplasmic domain of the membrane-tethered transcription factors translocate to the nucleus, where they activate transcription of their targets. ATF6 activates genes involved in endoplasmic reticulum (ER) stress response (1); SREBP activates genes involved in lipid homeostasis (2); BBF2H7 activates genes involved in chondrogenesis (3, 4); OASIS activates genes involved in osteoblast differentiation (5).

Pathogenic missense variants in *MBTPS2* are associated with two distinct conditions. The first is the dermatological spectrum conditions ichtyosis follicularis with atrichia and photophobia (IFAP) with or without BRESHECK syndrome (OMIM 308205), keratosis follicularis spinulosa decalvans (KFSD, OMIM 308800) and Olmsted syndrome (OS, OMIM 300918) (6). IFAP, KFSD and OS patients typically present with ichthyosis manifesting as thorn-like projections of follicular hyperkeratosis, absence of hair and photophobia (6–11). The second distinct clinical entity is X-linked recessive osteogenesis imperfecta type XIX (OI19, OMIM 301014), which has been described in few unrelated families carrying distinct pathogenic variants – c.1376A>G (p.Arg459Ser) and c.1515G>C (p.Leu505Phe); affected patients displayed moderate to severe OI with low bone mass, increased fracture frequencies, short stature, bowing of lower extremity long bones, scoliosis, pectal deformity and generalized osteopenia (12, 13). Despite clearly distinct clinical phenotypes, IFAP/KFSD/OS and OI can be caused by missense variants on neighbouring amino acid residues based on the secondary structure of S2P (14, 15).

Previously, we compared the molecular features of OI and IFAP/KFSD caused by pathogenic missense variants in *MBTPS2* (hereafter termed *MBTPS2*-OI and *MBTPS2*-IFAP/KFSD, respectively) by RNA-sequencing-based transcriptome profiling of control and patient-derived primary fibroblasts (14). The analysis highlighted changes in the expression of genes involved in bone and cartilage development in *MBTPS2*-OI but not *MBTPS2*-IFAP/KFSD fibroblasts compared to healthy controls; these genes included Dickkopf WNT Signaling Pathway Inhibitor 1 (*DKK1*), Vascular Endothelial Growth Factor A (*VEGFA*) and A Disintegrin and Metalloproteinase with Thrombospondin Motifs 12 (*ADAMTS12*). Furthermore, genes regulated by SREBP were more strongly downregulated in *MBTPS2*-OI than in *MBTPS2*-IFAP/KFSD fibroblasts, coupled with alterations in the relative abundance of fatty acids in *MBTPS2*-OI fibroblasts. A downregulation in the transcript levels of *COL4A1* and *COL4A2* and reduction in deposition of type IV collagen protein in the extracellular matrix (ECM) by both *MBTPS2*-OI and *MBTPS2*-IFAP/KFSD fibroblasts *in vitro* were observed (14).

More recently, in a male proband presenting *in utero* with features of a non-lethal skeletal dysplasia, exome sequencing using a brittle bone dysplasia panel found a hemizygous *MBTPS2* variant of unknown significance (VUS) – c.516A>C, p.Glu172Asp (NM_015884.4, NP_056968.1). No other variants relevant to the clinical phenotype were identified.

In an earlier study, an *MBTPS2* construct encoding the mutant p.Glu172Asp protein was coincidentally cloned without prior knowledge of this variant being expressed in patients, and overexpressed in Chinese Hamster Ovary cells deficient in *MBTPS2* (CHO-M19). The authors characterized the then-newly identified S2P protein and showed that it contains the HE*XX*H consensus sequence that constitutes the metal-binding site in metalloproteases; replacement of glutamic acid in the HE_172_ *XX*H motif with either alanine (p. Glu172Ala) or glutamine (p. Glu172Gln) completely abolished protease activity of S2P, while replacement with aspartic acid (p.Glu172Asp) caused significantly diminished S2P activity (16), which suggests that the variant identified in our proband can cause a detrimental effect. However, the pathogenicity of p.Glu172Asp in causing OI cannot be inferred from the data acquired using the CHO-M19 cells. This prompted us to extrapolate the knowledge acquired in our previous study on the unique molecular signature of *MBTPS2*-OI *in vitro* (14) to characterize the pathogenicity of this novel *MBTPS2* variant in causing severe OI.

## 2 Material and method

### 2.1 Proband’s clinical and genetic investigations

We studied a male fetus from a pregnancy in which ultrasound scans in the first trimester and at gestational week 20 + ^2^ raised the suspicious of a skeletal dysplasia. The aborted fetus at gestational week 21 + ^5^ was investigated with radiological and histological examinations. Genetic analysis followed by segregation analysis in the family were performed by exome and Sanger sequencing, respectively. The family gave informed consent for performing clinical and genetic investigations and consented to the publication of clinical photographs. This study was conducted according to the Declaration of Helsinki and approved by Swiss Ethics Committee (KEK-ZH-Nr. 2019-00811).

### 2.2 Cell culture

Primary fibroblast cultures were established from the umbilical cord of the proband (*MBTPS2* c.516A>C, p.Glu172Asp) and from punch skin biopsies obtained from two patients with *MBTPS2*-OI (c.1376A>G, p.Arg459Ser and c.1515G>C, p.Leu505Phe) (12). For ethical reasons, fibroblasts from punch skin biopsies of healthy children or from healthy fetuses could not be obtained. Therefore, fibroblasts established from the foreskin of three healthy children (termed hereafter as control children) and a fetus affected by glycogen storage disease type II without any skeletal abnormalities (termed control fetal) were used as controls. Informed consent of the patients or their parents were obtained in accordance with the requirements of the local ethics committees of the referring physicians.

Cells were cultured in Dulbecco Modified Eagle’s Medium (Gibco, 31966-021) supplemented with 10% fetal bovine serum (FBS, Gibco, 10270-106) and 1× antibiotic-antimycotic comprising 100 U/ml penicillin, 100 μg/ml streptomycin and 0.25 μg/ml Amphotericin B (Gibco, 15240062) at 37°C and 5% CO_2_.

### 2.3 Sanger sequencing

Sanger sequencing was performed to confirm the *MBTPS2* c.516A>C variant in the proband. DNA was extracted from control and proband-derived fibroblasts using QuickExtract DNA Extraction Solution (Lucigen, QE09050). PCR was performed with Phusion High-Fidelity DNA Polymerase (New England Biolabs, M0530) and primers flanking the region of interest (Forward: 5’-AGTTTGCAGAACATTCAGCTTG-3’, Reverse: 5’-CACTAGGCAATAGCCGCAGA-3’). The PCR products were subjected to Sanger sequencing using BigDye Terminator v3.1 Cycle Sequencing Kit (Applied Biosystems, 4337455) and analysed on the 3500-Genetic Analyzer (Applied Biosystems).

### 2.4 Transcriptional analyses

Candidate genes were selected based on the results obtained in our previous study (14) for quantification by quantitative reverse transcription polymerase chain reaction (qRT-PCR). Specifically, we quantified the transcript levels of Stearoyl-CoA Desaturase (*SCD*), Fatty Acid Desaturase 1 (*FADS1*) and Fatty Acid Desaturase 2 (*FADS2*) which are involved in fatty acid metabolism, and Dickkopf WNT Signaling Pathway Inhibitor 1 (*DKK1*), Vascular Endothelial Growth Factor A (*VEGFA*) and A Disintegrin and Metalloproteinase with Thrombospondin Motifs 12 (*ADAMTS12*) involved in bone development.

Total RNA was extracted from the fibroblasts upon reaching 90-100% confluency in a T75 flask using the RNeasy Plus Mini Kit (QIAGEN, 74134). RNA quality was checked by measuring the ratio of absorbance at 260nm and 230nm using the NanoDrop ND-1000 spectrophotometer (Thermo Scientific). Total RNA was reverse transcribed to produce cDNA using the High-Capacity RNA-to-cDNA Kit containing random octamers and oligo(dT)16 (Applied Biosystems, 4387406) following the manufacturers’ instructions. The cDNA was diluted to 3 ng/μl with nuclease-free water (Thermo Scientific, J71786) and used as template for qRT-PCR using TaqMan assays (**Table 1**) on a QuantStudio 7 Pro RT-PCR machine (Applied Biosystems). The transcript levels were calculated using the 2^-ΔCt^ method after normalization to the endogenous control genes *GAPDH*, *IPO8* and *TBP*. The experiment was repeated in four independent replicates.

**Table 1:** Taqman gene expression assays used for qRT-PCR.

Gene	Name	Assay ID
*TBP*	TATA-box binding protein	Hs00427620_m1
*IPO8*	Importin 8	Hs00914057_m1
*GAPDH*	Glyceraldehyde-3-phosphate dehydrogenase	Hs02786624_g1
*DKK1*	Dickkopf WNT Signaling Pathway Inhibitor 1	Hs00183740_m1
*VEGFA*	Vascular Endothelial Growth Factor A	Hs00900055_m1
*ADAMTS12*	A Disintegrin and Metalloproteinase with Thrombospondin Motifs 12	Hs00917098_m1
*SCD*	Stearoyl-CoA desaturase	Hs01682761_m1
*FADS1*	Fatty acid desaturase 1	Hs01096545_m1
*FADS2*	Fatty acid desaturase 2	Hs00927433_m1

### 2.5 Lipid analysis

Cells were cultured to 90-100% confluency in a T75 flask and collected by trypsinization with 0.05% Trypsin-EDTA (Gibco, 25300-054) and centrifugation at 930 rpm for 5 minutes. The cell pellets were washed once with Dulbecco’s Phosphate Buffered Saline (DPBS, Gibco, 14190144) and pelleted by centrifugation at 8000 rpm for 5 minutes at 4°C. The cellular fatty acids were determined by gas chromatography coupled to a tandem mass spectrometer (GC-MS/MS, Thermo Scientific, TSQ 8000), according to a previously described method (17). In brief, each fibroblast sample was spiked with internal standards comprising [d_33_]-heptadecanoic acid and [d_43_]-docosanoic acid; fatty acids were then extracted from the samples using a mixture of methanol and dichloromethane in a TissueLyser II following the manufacturer’s protocol (QIAGEN). The organic phase was transferred to a glass tube and derivatized with acetyl chloride at 95°C for one hour. The resulting fatty acid methyl esters were purified by liquid-liquid extraction with hexane, dried under a flow of nitrogen and dissolved in heptane. The samples were then injected into the GC-MS/MS system and recorded using selective reaction monitoring (18). Quality control is performed by checking the intensity and peak area of the internal standards. Fatty acid quantification is performed using the Xcalibur ICIS peak integration and detection algorithm (Thermo Scientific), with calibration using standards for each fatty acid at six concentrations within the physiologically-relevant range.

### 2.6 Immunocytochemistry

Fibroblasts were plated on glass coverslips and cultured in 6-well plates at a density of 4 × 10^5^ cells per well. The cells were cultured in macromolecularly crowded medium comprising 37.5 mg/ml Ficoll PM70 (Sigma, F2878), 25 mg/ml Ficoll PM400 (Sigma, F4375), 0.5% FBS and 1× antibiotic-antimycotic in DMEM. L-ascorbic acid (Sigma, A4544) was freshly prepared and added to the media at 50 μg/ml daily to promote ECM protein deposition. After four days, cells were fixed in 4% paraformaldehyde (Thermo Scientific Chemicals, J61899) for 10 minutes at room temperature and washed three times with DPBS.

For immunocytochemistry, the coverslips were blocked with 1% bovine serum albumin (Sigma, A3912) in DPBS for one hour at room temperature. The samples were not permeabilized to detect only ECM proteins. After blocking, the coverslips were incubated overnight at 4°C with primary antibodies against type I collagen (Abcam, ab90395, 1:500; RRID : AB_2049527), type IV collagen (Developmental Studies Hybridoma Bank, M3F7, 1:25; RRID : AB_528167), type V collagen (Millipore, AB763P, 1:50; RRID : AB_2083077) or integrin α_2_β_1_ (Millipore, MAB1998Z, 1:100; RRID : AB_94501). For co-staining of samples for misfolded collagen chains and type I collagen, coverslips were co-incubated overnight at 4°C with 20 µM collagen hybridizing peptide conjugated to Cyanine3 (R-CHP, 3Helix) and antibodies against type I collagen (Abcam, ab90395, 1:500; RRID : AB_2049527). The coverslips were then washed three times with PBS and incubated for one hour at room temperature with Alexa Fluor 488-conjugated anti-mouse IgG antibodies (Invitrogen, A11017) or Alexa Fluor 488-conjugated anti-rabbit IgG antibodies (Invitrogen, A11008). Finally, samples were washed thrice with PBS and once with distilled water and mounted onto glass slides using a mounting solution containing the nuclear stain 4′,6-diamidino-2-phenylindole (Sigma, F6057). Imaging was performed on a Leica DMi8 microscope with a Leica DFC3000 G camera.

## 3 Results

### 3.1 Proband’s clinical and genetic findings

**Figure 1:**
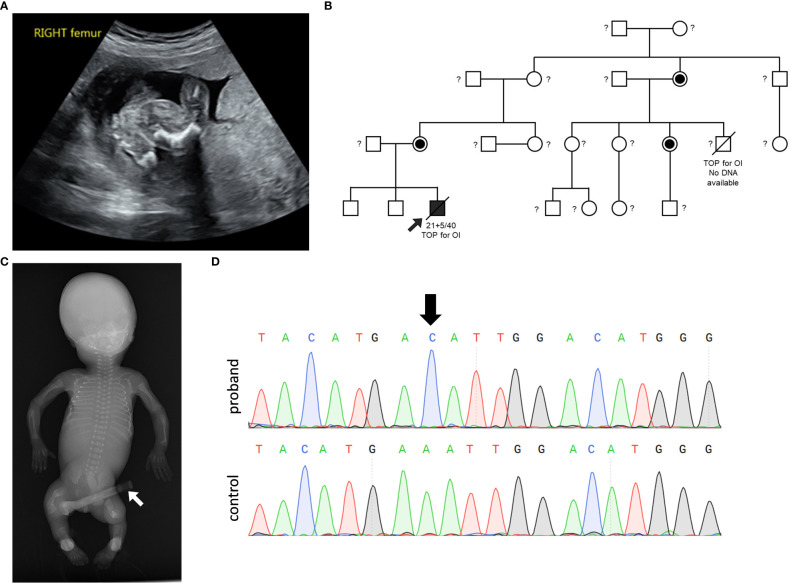
Clinical and genetic information. **(A)** Ultrasound at gestational week 20 of the right femur demonstrating shortened and bowed femur. **(B)** Pedigree of the family demonstrating X-linked inheritance of the OI phenotype and *MBTPS2* variant. The male proband is depicted with an arrow. A termination of pregnancy (TOP) due to the presentation of OI phenotype in a male cousin is notable; DNA from the affected male cousin was not available for genetic testing. Female carriers of the VUS are depicted by the half-filled-in circle symbol. Untested individuals are indicated with a question mark ‘?’ symbol. **(C)** Anterior-Posterior radiograph of the male proband shows reduced bone density, multiple rib and long bone fractures, and broad deformed femora as a result of previous healed fractures. The device shown by a white arrow represents an umbilical cord clamp. **(D)** PCR-sequencing of *MBTPS2* confirmed the presence of the c.516A>C variant in the proband, as depicted by the black arrow on the chromatogram.

The first trimester scan at 12 weeks gestation reported a hypoplastic nasal bone and a resultant chorionic villous sampling reported a normal male CGH microarray. Ultrasound scan at 20 + ^2^ weeks gestation demonstrated shortening of all long bones and bowing of the femurs and tibiae, a normal ribs and chest circumference (**Figure 1A**). The family history was notable for a termination of pregnancy for “brittle bone disease” in a maternal aunt (**Figure 1B**). The mother was of normal stature, had normal sclerae and dentition and had no history of fractures. The pregnancy was terminated at 21 + ^5^ weeks gestation. Post-delivery examination revealed micromelia particularly of the lower limbs associated with bowing of the right thigh and severe (almost 90 degrees) angulation of the right and left tibiae anteriorly but no talipes, both radii appeared prominent and angulated close to the wrists, a narrow thorax, and deficient ossification of much of the calvarium.

Examination on autopsy correlated with radiological investigation (**Figure 1C**) demonstrating a partially ossified calvarium with very thin and almost translucent frontal and parietal bones measuring <1mm in thickness, thirteen pairs of ribs with multiple rib fractures of varying ages, a normal spine and short and bent femurs and tibiae. Histological examination of the bones was consistent with healed and healing fractures. In summary, the suspected clinical diagnosis of OI was confirmed.

Genetic analysis performed with a brittle bone dysplasia panel testing using a exome sequencing backbone identified the *MBTPS2* c.516A>C, p.Glu172Asp VUS in the proband and subsequent segregation analysis demonstrated that none of the proband’s non-affected brothers carried the variant and both the mother of the proband as well as the maternal aunt whose pregnancy was also terminated based on the suspicion of carrying a brittle bone disease were heterozygous for the variant (**Figure 1D**).

### 3.2 Differentially expressed genes involved in bone and cartilage development

**Figure 2:**
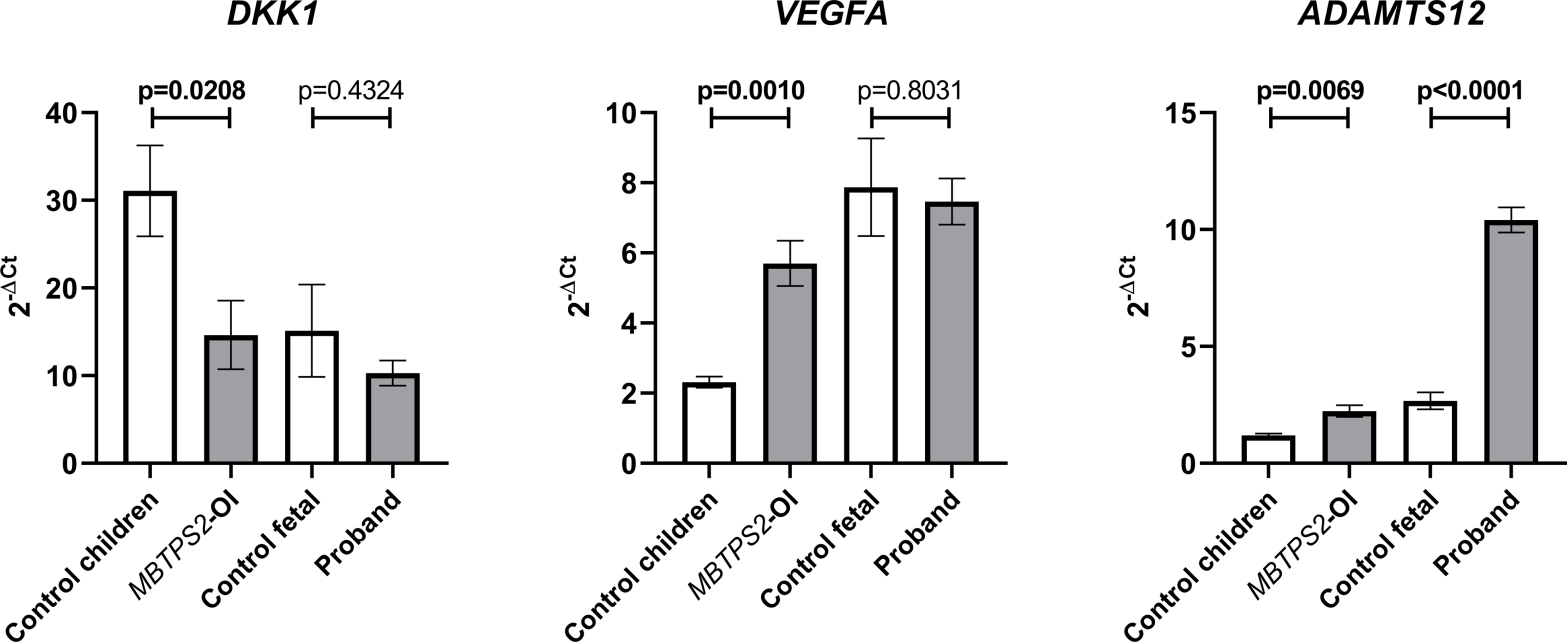
Expression levels of genes involved in skeletal development. Transcript levels for Dickkopf WNT Signaling Pathway Inhibitor 1 (*DKK1*), Vascular Endothelial Growth Factor A (*VEGFA*) and A Disintegrin and Metalloproteinase with Thrombospondin Motifs 12 (*ADAMTS12*) were measured in four independent replicates per subject by qRT-PCR. A Welch’s t-test was performed for statistical analysis. Data are reported as mean ± SEM. Significant p-values < 0.05 are indicated in bold.

*DKK1*, *VEGFA* and *ADAMTS12*, which are genes involved in bone or cartilage homeostasis, were previously identified amongst the top hits of differentially expressed genes (DEGs) in *MBTPS2-*OI compared to controls in our transcriptomics studies (14). Therefore, the expression of these genes were quantified by qRT-PCR and compared between control children and *MBTPS2*-OI fibroblasts and between the control fetal and proband fibroblasts. We observed significant downregulation in the expression of *DKK1* and significant upregulation of *VEGFA* and *ADAMTS12* in *MBTPS2*-OI patient fibroblasts, recapitulating our previous findings. In proband-derived cells, the expression of *ADAMTS12* was also strongly upregulated; a trend for downregulation of *DKK1* was observed but was statistically not significant. No difference in the expression of *VEGFA* was found between control fetal and proband fibroblasts (**Figure 2**).

### 3.3 Fatty acid metabolism

**Figure 3:**
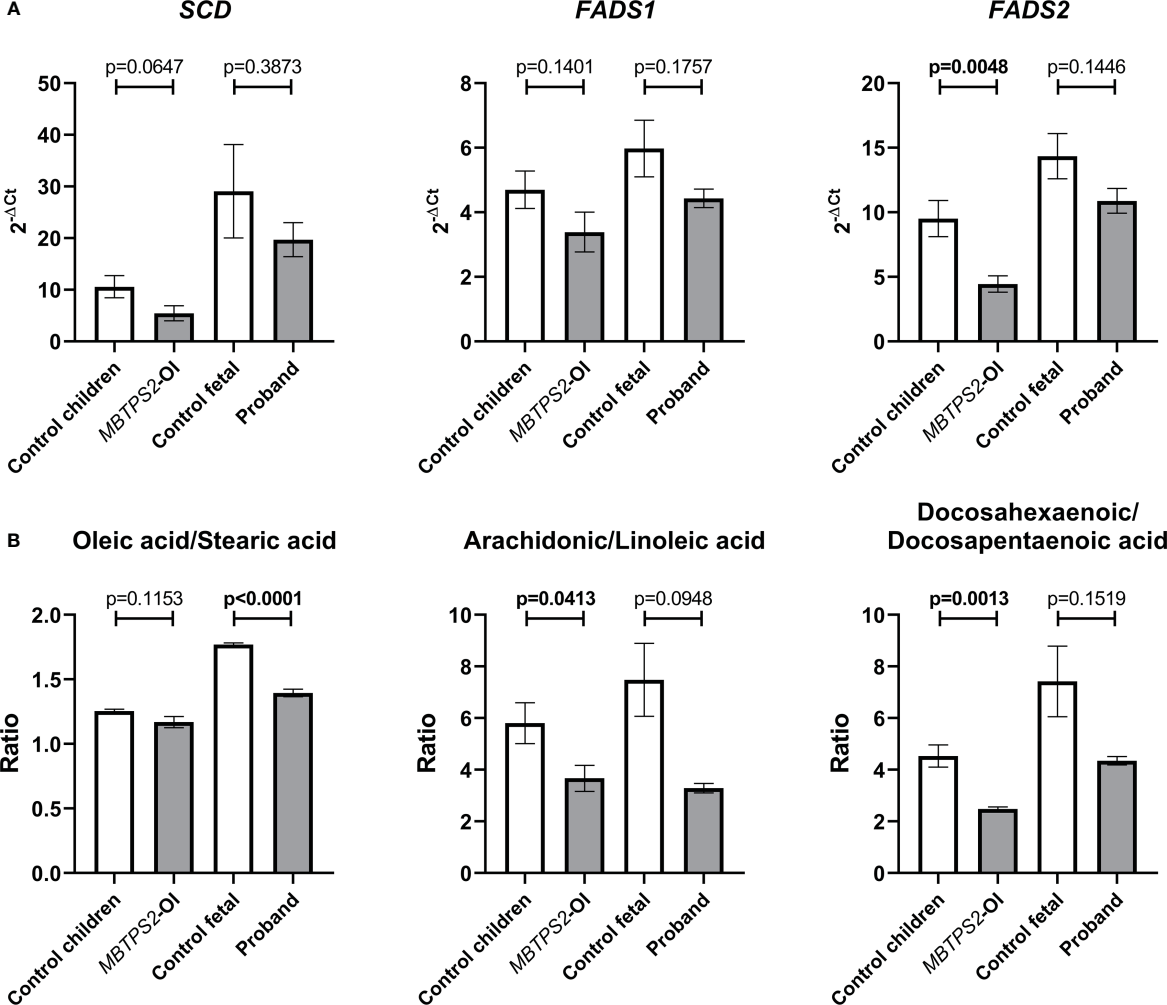
Assessment of fatty acid metabolism by quantification of transcript levels of genes involved in fatty acid metabolism and relative abundance of cellular fatty acids. **(A)** Expression levels of SREBP-dependent genes involved in fatty acid metabolism, namely Stearoyl-CoA Desaturase (*SCD*), Fatty acid desaturase 1 (*FADS1*) and Fatty acid desaturase 2 (*FADS2*) were measured in four independent replicates per subject by qRT-PCR. A Welch’s t-test was performed for statistical analysis. Data are reported as mean ± SEM. Significant p-values < 0.05 are indicated in bold. **(B)** Cellular contents of oleic acid, stearic acid, arachidonic acid, linoleic acid, docosahexaenoic acid and docosapentaenoic acid were measured in three independent replicates per subject by GC-MS/MS and their relative abundance are expressed as ratios. A Welch’s t-test was performed for statistical analysis. Data are reported as mean ± SEM. Significant p-values < 0.05 are indicated in bold.

The SREBP-target genes *SCD*, *FADS1* and *FADS2* are involved in fatty acid metabolism and were significantly downregulated in *MBTPS2*-OI (14). Stearoyl-CoA desaturase (*SCD*) converts stearic acid to oleic acid, whereas Δ^5^-desaturase (*FADS1*) and Δ^6^- desaturase (*FADS2*) are involved in the synthesis of arachidonic acid from linoleic acid and of docosahexaenoic acid (DHA) from docosapentaenoic acid (DPA). In the current study, a trend towards the suppression of *SCD*, *FADS1* and *FADS2* was observed in *MBTPS2*-OI compared to control children and also in the proband compared to control fetal fibroblasts; statistical significance was only seen in the suppression of *FADS2* in *MBTPS2-*OI (**Figure 3A**). Nevertheless, analyses of the relative abundance of intracellular fatty acids by GC-MS/MS suggested reduced activities of the enzymes encoded by *SCD*, *FADS1* and *FADS2*. A significant decrease in the oleic acid to stearic acid ratio was observed in the proband fibroblasts compared to the fetal control. The ratios of arachidonic acid to linoleic acid and DHA to DPA were significantly reduced in *MBTPS2*-OI, similar trends were observed for the proband compared to fetal control (**Figure 3B**).

### 3.4 Protein deposition in the ECM

**Figure 4:**
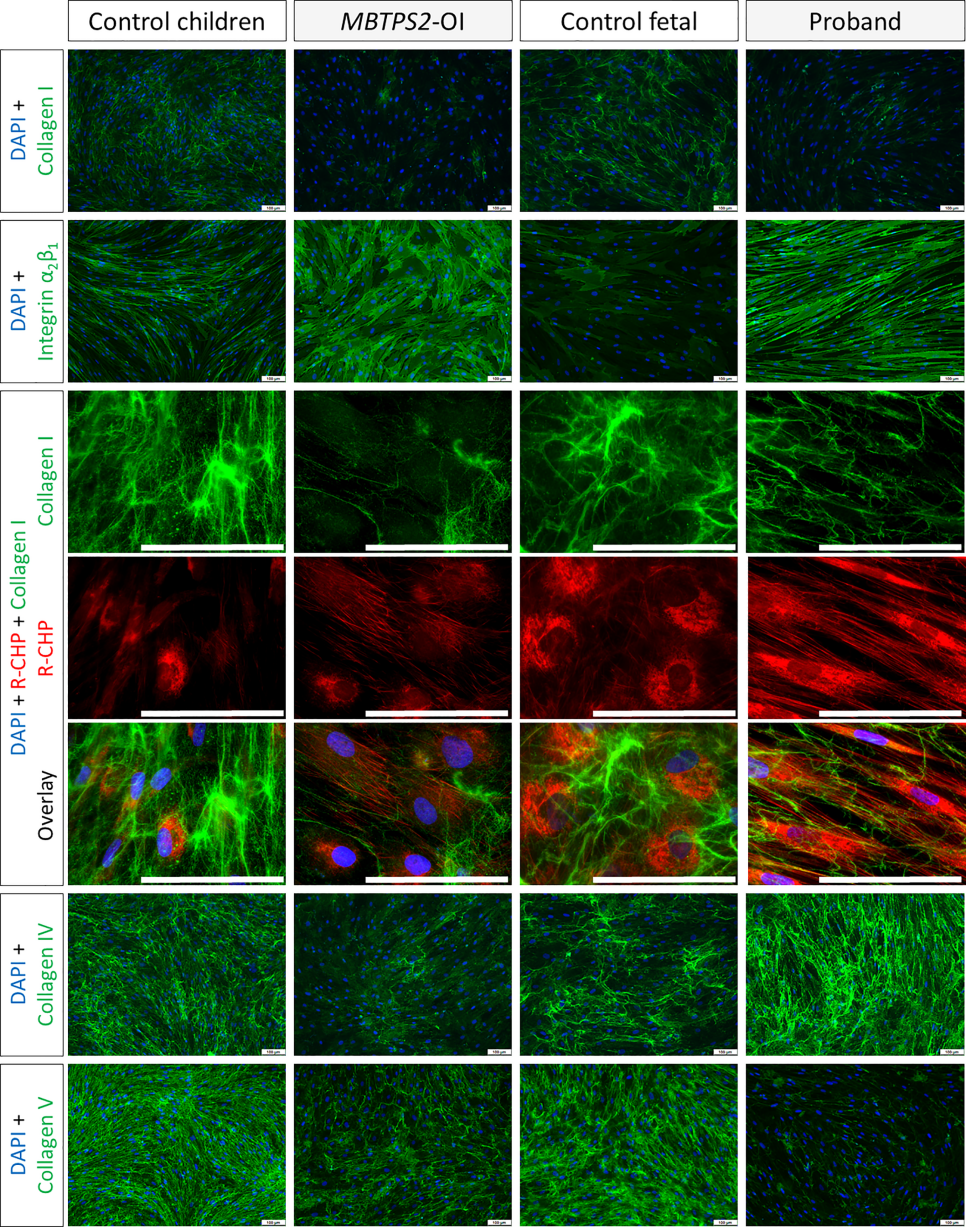
Extracellular matrix protein deposition Immunocytochemistry analyses were performed on fibroblasts cultured under macromolecularly crowded conditions and given fresh ascorbic acid daily for four days. The cell nuclei are depicted in blue; type I, IV and V collagens and integrin α_2_β_1_ are detected by immunostaining and depicted in green. Misfolded collagens that are deposited in the ECM are detected by hybridization with R-CHP and depicted in red, particularly in long, parallel fibrous-like staining patterns. Representative images from one subject per group are shown. Scale bar (white) = 100 µm.

By immunocytochemistry, we determined differences in the deposition of ECM proteins by fibroblasts obtained from control children, *MBTPS2-*OI patients, fetal control and the proband *in vitro*. The deposition of type I collagen in the ECM by *MBTPS2-*OI fibroblasts was reduced compared to control children fibroblasts; similarly, fibroblasts from the proband deposited less type I collagen than that from the fetal control. A monoclonal antibody that inhibits integrin α_2_β_1_-mediated cell adhesion to collagen and laminin was used to detect the free epitope on integrin α_2_β_1_ that is not engaged in collagen or laminin binding. Consistent with a reduction in type I collagen deposition by *MBTPS2-*OI and proband fibroblasts, an increase in free integrin α_2_β_1_ was detected in these fibroblasts compared to their respective controls. Furthermore, a single-stranded collagen mimetic peptide that has a strong binding affinity for unfolded triple helical chains of all types of collagen (R-CHP, 3Helix) due to its shared Gly-X-Y repeating sequence with natural collagen structure (19) was used to detect misfolded collagen deposited in the ECM. Although stronger R-CHP staining could be seen in fetal cells compared to non-fetal cells in general, more intensive staining was observed in the *MBTPS2-*OI and proband fibroblasts compared to their respective developmental age-matched fibroblasts. In particular, fibrous-like R-CHP staining could be seen in *MBTPS2-*OI and proband fibroblasts suggesting that the fibrous collagen deposited by these cells are misfolded (**Figure 4**). The deposition of type IV collagen in the ECM was reduced in *MBTPS2-*OI fibroblasts compared to control children fibroblasts, in agreement with our previous observations (14). However, the opposite trend was seen in the proband fibroblasts, which deposited more type IV collagen than the control fetal cells. Type V collagen deposition was reduced in both *MBTPS2-*OI and proband fibroblasts in comparison to their respective controls (**Figure 4**).

## 4 Discussion

In the last years, the global assessment of biomolecules using omics technologies including genomics, transcriptomics, proteomics and metabolomics has allowed the identification of biomarkers or molecular signatures associated with certain diseases, thus providing insights in their underlying path mechanisms. The application of Next-Generation Sequencing (NGS) in genomics has undoubtedly facilitated the identification of disease-causing variants and improved the diagnostics of heritable disorders in the last decade. However, reliable interpretation of genetic variants proved to be complex due to the presence of single nucleotide variants without known pathogenic effect (20) and limitations of *in silico* prediction tools (21, 22). As a result, NGS cannot offer an accurate diagnosis for every case with presumable genetic etiology and often leads to identification of variants of unknown significance (VUS) in known disease genes or in candidate genes that await proof of their clear association with a disease (20, 23). Pleiotropic effects of one gene on multiple seemingly unrelated phenotypic traits further complicate the assessment of genetic variants. Correct interpretation of exome or genome sequencing results can often only be achieved by functional studies. The integration of knowledge on multiple omics level is a useful approach to understand diseases at the molecular level and allows functional studies to be established to confirm the interpretation of genetic variants (23–25).

Investigations based on the expression of mutant *MBTPS2* constructs in CHO-M19 cells provided valuable knowledge on the catalytic sites of the S2P enzyme (16). However, such an approach to investigate novel variants require extensive time to clone and express mutant constructs and the availability of cells lacking endogenous expression of the gene of interest for recessive variants. The approach is further limited by the downstream analyses that can be performed to infer pathogenicity of the variants on a disease.

In this study, we applied our knowledge on the molecular signature of *MBTPS2*-OI that we previously gathered via transcriptome, lipidome and ECM proteome analyses of patient-derived fibroblasts to infer the pathogenicity of the novel MBTPS2 p.Glu172Asp variant in a severe case of Osteogenesis imperfecta. In our earlier study, transcriptomics analysis was performed to identify genes whose expression are changed in *MBTPS2*-OI but unaltered in *MBTPS2*-IFAP/KFSD to serve as a molecular signature that distinguishes OI from IFAP/KFSD. We found changes in the expression of genes involved in bone and cartilage development (downregulation of *DKK1*, upregulation of *VEGFA* and *ADAMTS12*) and SREBP-target genes (downregulation of *SCD*, *FADS1* and *FADS2*) in *MBTPS2*-OI patient-derived fibroblasts. Here, we demonstrate in the proband-derived fibroblasts a significant increase in the expression of *ADAMTS12*, but could not detect significant changes in the expression of *DKK1* and *VEGFA*. This inconsistency might be explained by the relationship between developmental age and the expression of these genes, in which we saw a lower expression of *DKK1* and elevated expression of *VEGFA* in the control fetal cells compared to control children cells, thereby masking further down and upregulation of the genes, respectively. In more detail, the upregulation of *VEGFA*, which we saw specifically in *MBTPS2*-OI in our previous study, was interpreted as a compensatory mechanism of *MBTPS2*-OI cells to favour bone formation. In the proband and control fetal fibroblasts *VEGFA* is similarly expressed, but compared to control children it is overexpressed. Thus, during development there might be a constitutional overexpression of *VEGFA* to promote bone formation via endochondral ossification. It has been shown that DKK1 switches mesenchymal stem cell differentiation from osteoblastogenesis to adipogenesis (26–28). In our previous study, the downregulation of *DKK1* in *MBTPS2*-OI cells - similarly to the overexpression of *ADAMTS12* and *VEGFA* - was interpreted as a mechanism in *MBTPS2*-OI cells to counteract the inhibitory effect of DKK1 on osteoblast differentiation and bone formation, and its promotion of osteoclastogenesis (27) and adipogenesis, thus as an attempt to rescue bone formation. We speculate that during development this compensatory “switch-off” mechanism might be constitutively activated in fetal cells to assure in the fetus the equal maturation of other tissues, in particular the adipose tissue. A trend towards downregulation of *SCD*, *FADS1* and *FADS2* and alterations in the relative abundance of fatty acids was observed in the proband fibroblasts, which highlights perturbations in fatty acid metabolism in the proband similar to that observed in *MBTPS2*-OI patients. In this study, we expanded the molecular characterization of *MBTPS2*-OI and proband fibroblasts by probing several ECM proteins deposited by the cells *in vitro*. Similar changes in type I and V collagen and free integrin α_2_β_1_ receptor could be observed in *MBTPS2*-OI and the proband compared to their age-matched controls. Changes in type IV collagen deposition was inconsistent; this suggests that type IV collagen unlikely plays a role in the pathogenesis of *MBTPS2*-OI, which is in line with the lack of reports on its involvement in OI to date.

Our study is limited by the small sample size due to the rare nature of this genetic form of OI. With the identification of more *MBTPS2*-OI patients and their molecular characterization via multiple omics approaches in future, the molecular signature of this disease will be fine-tuned to better understand the disease path mechanisms and prove the pathogenicity of novel genetic variants. Furthermore, our approach to inferring pathogenicity of novel genetic variants requires the use of patient fibroblasts which are not always available and is restricted for research purposes only. We currently address these limitations by working to identify biomarkers in blood or urine of a larger patient cohort to allow for more rapid and less invasive diagnostic approaches to validate VUSs in future.

Nevertheless, our study suggests that fatty acid metabolites and type I collagen and its interaction protein partners are more robust indicators of *MBTPS2-*OI pathology. This is likely due to a direct involvement of S2P in regulating SREBP transcription factor activity, resulting in perturbations in fatty acid metabolism as a primary consequence of *MBTPS2* variants. In addition, type I collagen is the major organic component of the ECM in bone; type V collagen is present in small amounts within heterotypic type I collagen fibrils and regulates collagen fibrillogenesis. Dysregulation in types I and V collagen in *MBTPS2*-OI suggests a significant effect on bone formation as a result of functional consequences of certain variants in the *MBTPS2* gene, although the exact mechanism leading to the reduction in quantity and/or quality of type I and V collagens in *MBTPS2*-OI remains to be elucidated.

In conclusion, we find that the overlapping trends in the molecular characteristics of fibroblasts derived from the proband and known *MBTPS2*-OI patients at the transcript, protein and relative abundance of fatty acid levels supports biological importance and the pathogenicity of *MBTPS2* c.516A>C (p.Glu172Asp) in OI. However, we would like to emphasize that the data is based on primary cells from a single proband and interpretation of the statistical significance should be taken with caution. Disease models for *MBTPS2*-OI are currently being established to facilitate more thorough investigations and enable the discovery of more accurate and robust molecular signatures to infer pathogenicity of newly reported variants in future.

## Data availability statement

The data presented in the study are deposited in the Zenodo repository, accession number 7935632.

## Ethics statement

The studies involving human participants were reviewed and approved by Swiss Ethics Committee. Written informed consent to participate in this study was provided by the participants’ legal guardian/next of kin. Written informed consent was obtained from the individual(s), and minor(s)’ legal guardian/next of kin, for the publication of any potentially identifiable images or data included in this article.

## Author contributions

CG, MR, and PJL contributed to the conceptualization of the project. JP provided the clinical description and fibroblasts from the proband. GM and PJL contributed to the gene expression and immunocytochemistry analyses. PS contributed to the fatty acid analyses. TN contributed to cell culture work. GM and PJL drafted the manuscript. All authors contributed to the article and approved the submitted manuscript.
